# With confidence comes success: an exploration of high school students’ mental health education from the perspective of self-efficacy theory

**DOI:** 10.3389/fpsyg.2026.1667290

**Published:** 2026-01-26

**Authors:** Peng Su, Xiaolong Li

**Affiliations:** 1 The Faculty of Education, Southwest University, Chongqing, China; 2 The Faculty of Education, Henan University, Kaifeng, Henan, China

**Keywords:** high school students, home-school collaboration, mental health, mental health education, self-efficacy

## Abstract

**Introduction:**

High school is a critical developmental period in which adolescents’ psychological functioning and social adaptability are rapidly reconfigured. However, mental health education in Chinese senior high schools often lacks systematic approaches in preventive intervention, pedagogical depth, and the cultivation of students’ psychological potential. Self-efficacy theory offers an informative lens for improving school-based mental health education.

**Methods:**

Situated in the context of senior high school mental health education, this qualitative study used semi-structured interviews and grounded theory to examine how self-efficacy is generated and evolves across learning and everyday-life contexts, and how it relates to mental health outcomes. Participants were recruited from a senior high school in Zhengzhou. Twenty-three students in Grades 10–11 were interviewed. Data were analyzed using an iterative grounded-theory coding procedure to develop core categories and an integrative relational model.

**Results:**

The findings indicate a close and dynamic association between self-efficacy and adolescents’ mental health. Success and failure experiences, as well as feedback in key situations—such as academic stress, peer interactions, and self-evaluation—shape both moment-to-moment fluctuations and the longer-term development of self-efficacy. These self-efficacy processes are linked to emotional stability, coping patterns, and overall psychological adjustment.

**Discussion:**

We propose a self-efficacy–oriented mechanism for mental health education and outline a coordinated support pathway spanning school and family. The findings provide theory-grounded and actionable implications for shifting school mental health work from predominantly reactive remediation toward development-oriented prevention, and for strengthening systematic home–school collaboration in practice.

## 1 Introduction

### 1.1 Overview of the current status of mental health education for high school students

The 2023 Blue Book on Mental Health in China shows that the mental health problems of the adolescent population are becoming increasingly prominent, with high school students experiencing particularly severe psychological issues and a depression detection rate exceeding 40% (Rao et al., 2024). In April 2025, the National Mental Health Development Report of China (2023–2024) was officially released, showing a distinct age stratification of mental health problems, with high school students reaching a peak in depression levels. The above research results all indicate that high school students have frequent mental health problems and are at the bottom of their adolescent mental health level. The high school stage, as a critical period for individual physiological development and psychological maturity, faces multiple challenges such as physiological development, academic pressure, and interpersonal relationships. At the same time, the psychological state of high school students during this stage presents a “semi mature and semi childish” characteristic, which is prone to psychological health problems such as anxiety and depression due to academic setbacks, emotional confusion, or self-awareness biases (Liu et al., 2018).

Therefore, high school mental health education is not only an important component of the education system, but also an important means to prevent psychological problems among high school students and tap into their psychological potential. Effective implementation of mental health education is an important cornerstone for improving the personality of high school students and promoting their comprehensive development. The previous concept of mental health focused on psychological symptoms, but with the development of positive psychology, a complete mental health state should also include the presentation of positive emotions, as well as an individual’s positive functioning at the psychological and social levels (Tian and Cai, 2024). Although positive psychology has expanded the connotation of mental health and provided a certain theoretical basis for high school mental health education, this concept has not yet been implemented in educational practice. Specifically, on the one hand, the effectiveness of transforming the concept of adolescent mental health education is insufficient. At the school level, there is a widespread trend of psychological health education that only solves problems after discovering them. However, a psychological health education and training system that replaces intervention with prevention and cultivates positive psychological qualities in adolescents to reduce the occurrence of psychological problems has not been formed (Reavley and Jorm, 2010). At the family level, parents have misconceptions about the mental health issues of high school students, making it difficult to prevent, identify, and intervene in the mental health problems of adolescents. On the other hand, research on mental health education often starts from the perspective of educators, emphasizing their understanding and reflection on mental health education, and rarely starting from the perspective of high school students to explore the needs of educational objects for mental health education.

### 1.2 Self efficacy is the core regulatory mechanism of mental health

Self efficacy refers to the level of confidence that individuals have in their ability to use the skills they possess to complete a certain task. It often affects behavior choices, motivation, effort, cognitive processes, and emotional processes, playing a central role in personal cognitive processes (Bandura, 2001). Through reviewing previous studies, the impact of self-efficacy on adolescent mental health presents a dual pathway mechanism of immediate and long-term effects.

At the immediate effect level, self-efficacy has a significant impact on high school students’ task execution. High school students are frequently exposed to task-based situations such as academic assessments and subject competitions, which not only constitute significant pressure loads, but also carry multiple functional meanings such as academic achievement and self-identity. Once encountering unexpected setbacks during the task process, an individual’s self-efficacy will undergo a sharp change, which can trigger an escalation of negative emotions (Pekrun, 2006). Thus affecting the individual’s current level of mental health. At the same time, negative emotions generated during the task can also lead to excessive use of limited cognitive resources, resulting in difficulty or poor completion of the task (Lievore et al., 2024). Furthermore, it triggers a negative emotional behavioral cycle, reduces individual self-efficacy, and has a negative impact on mental health (Orbach et al., 2020). In task contexts, self-efficacy has a significant impact on the mental health status of adolescents through negative emotions. Clarifying this mechanism of influence has important practical implications for adolescents to overcome the negative effects of changes in self-efficacy during the task process.

On the long-term effect level, self-efficacy plays a core regulatory role in individual psychological regulation and achievement behavior, and is an important protective factor for mental health (Li et al., 2013). According to social cognitive theory, an individual’s health behavior is not driven by internal psychological forces, nor is it completely influenced by external environmental stimuli. Instead, it is a product of the interaction between the environment, behavior, and personal cognition (Bandura, 1986). Its mechanism of action can be further explained as follows: individuals with high self-efficacy tend to adopt positive coping strategies in various situations, enhance cognitive ability and emotional regulation effectiveness through multiple success and failure experiences, and form a virtuous cycle of psychological health. A meta-analysis study on self-efficacy and mental health confirms that self-efficacy can positively affect positive factors of mental health, while negatively affecting negative factors of mental health (Guo and Li, 2025). It should be noted that age has a significant moderating effect on the relationship between self-efficacy and mental health, manifested in the stronger relationship between self-efficacy and positive factors of mental health in minors compared to adults (Li et al., 2019). Therefore, the high school stage is a critical period for cultivating self-efficacy. During this stage, students’ cognitive abilities have approached those of adults, but their self-identity is not yet stable. They are more sensitive to external evaluations and successful experiences, and the plasticity of educational interventions is stronger. Seizing this window of self-efficacy provides a starting point for the construction of high school mental health education.

After reviewing previous research, both theoretical and empirical evidence indicate that self-efficacy influences adolescent mental health from both immediate and long-term effects (Zhang et al., 2023). However, there are still two prominent issues in current research. First, many studies adopt a predominantly macro-level perspective, such as policy frameworks and institutional arrangements, while paying insufficient attention to the micro-level mechanisms through which mental health education operates in everyday school life. In practice, effective mental health education should be student-centered and enacted by educators, with an emphasis on preventing psychological difficulties and fostering positive psychological qualities. Second, existing work has largely advanced recommendations from the perspective of formal curricula and the construction of curriculum systems. Comparatively less is known about how non-curricular carriers of mental health education, including routine school practices and contextual supports, may produce sustained educational influence, and how a stable mechanism for home–school collaboration can be designed and implemented. To address these limitations, the present study pursues two interconnected objectives. The empirical objective is to use qualitative inquiry to elucidate the internal mechanisms linking self-efficacy to adolescent mental health, grounded in students’ lived experiences. The applied objective is to translate the empirically derived insights into structured intervention directions at both school and family levels, thereby offering actionable guidance for mental health education practice. Accordingly, we conduct semi-structured interviews to capture adolescents’ needs for mental health education and to clarify the ways self-efficacy is generated and transformed across learning and everyday contexts (Gueldner et al., 2020). Not only does it continue the student-centered needs revealed in the interview, but it also embeds the cultivation of self-efficacy into the entire process of mental health education through systematic construction of non curriculum carriers and home school collaboration mechanisms, forming a comprehensive intervention path from micro psychological mechanisms to macro educational ecology, exploring the psychological potential of high school students, and shaping their positive personality.

## 2 Methods

### 2.1 Participants

Participants were recruited from a senior high school in Zhengzhou, China. Recruitment announcements were delivered by the course instructor during regular mental health education classes for Grade 10 and Grade 11 students. Students who were interested in participating voluntarily registered their information. After registration, the school mental health teacher and the research team jointly reviewed the applicant list using predefined inclusion/exclusion criteria. To minimize potential psychological risk and ensure suitability for a non-clinical interview, students who showed abnormal results in the school’s annual mental health screening were excluded from participation. Eligible students were then contacted to schedule interviews, and all participants were assigned anonymized identifiers (S1–S23). Participation was entirely voluntary and had no impact on students’ course grades or access to school services; informed consent procedures were implemented prior to data collection.

### 2.2 Interview tools

In order to ensure the richness and effectiveness of the collected interview data, this study followed the following three principles when designing the interview outline. One is to design according to the requirements of the high school’s mental health teacher and the interviewed student’s homeroom teacher, to ensure that the interview will not have a negative impact on the students and to understand their basic mental health status. Secondly, when presenting interview questions, researchers choose to integrate various methods such as open, semi open, and closed formats. Open ended questions allow respondents to freely express their true thoughts; semi open-ended questions enhance ecological validity by simulating real-life situations; closed ended questions can be used to validate existing hypotheses or confirm new discoveries. Finally, this interview was a one-on-one semi-structured in-depth interview with high school students, conducted during their spare time and mental health classes. When determining the duration of an interview, it is not only necessary to consider the quality of the interview, but also to avoid awkward or lengthy situations during the interview process. Therefore, a standardized 20 min interview procedure was adopted (Yi-Jun et al., 2022).

This study focuses on the impact of self-efficacy on mental health and conducts interviews. The main questions in the interview outline are designed from two perspectives: immediate and long-term scenarios. Three main questions are designed for each perspective to form the overall framework of the interview outline. The following is the finalized interview outline. During the actual interview process, researchers will ask appropriate questions based on the feedback from the interviewees to obtain more accurate information.

1. Please use a few words to express your current learning and living situation.
2. What do you think a good mental health state should be.
3. Please describe in detail the specific process of encountering setbacks in a task (such as an exam, competition, etc.) and explain your emotional feelings at that time.
4. What were the ways you dealt with setbacks in the experience you just shared? How does this coping style affect your subsequent task performance?
5. What do you think you would do if you encountered a similar situation again.
6. Looking back at the past 6 months, which experiences have significantly boosted your confidence? Have these experiences affected your own confidence?
7. Based on your observation, what are the differences between high confidence and low confidence students in the following behaviors (learning attitude, stress coping, social conflict management)?
8. If you were to draw your confidence curve, which do you think is the most important, family support, teacher support, and peer support, and what are their respective proportions?
9. What do you think confidence means to you in life and study, and what kind of impact can it have?
10. Are there any other relevant experiences or important emotional feelings that need to be supplemented?

### 2.3 Interview process

The interview adopts a semi-structured format, with the theme of “The Impact of Self Efficacy on Mental Health.” Due to the mention of participants’ privacy during the interview and to enhance their sense of security, the interview was conducted in the counseling room of the mental health center to ensure that the interview process was not disrupted. The specific interview time is determined by the interviewee based on their spare time, and each person’s interview lasts about 20 min.

Researchers transcribe audio files word for word and match the transcribed text with the audio to ensure accuracy. Subsequently, the specific task information of the interviewees was deleted from the written records and assigned a code to each interviewee for organized data analysis in the future (Powney and Watts, 2018). The transcribed manuscript amounts to approximately 124,000 words.

## 3 Interview results

### 3.1 Data analysis

All interview recordings were transcribed verbatim and checked against the audio to ensure accuracy. Identifiable task-specific information was removed during anonymization, and each participant was labeled S1- S23 for subsequent analysis. The final corpus contained approximately 124,000 words. The transcripts were imported into NVivo 12.0 for data management and systematic coding (Edhlund and McDougall, 2018).

### 3.2 Reliability and validity testing

Unlike quantitative research, the reliability of qualitative research is difficult to evaluate using quantitative indicators. This study mainly adopts two methods to improve the reliability of data analysis: first, continuous iterative coding. Researchers have coded, analyzed, and re coded from their own perspectives, enhancing the validation of the analysis results. Secondly, it is reflection and discussion. The coding personnel and experts in this study regularly engage in in-depth discussions, reflecting and revising the research results based on the perspectives of the research members.

Structural factors may be an important factor affecting the validity of interviews (Liu, 2018). This study ensures interview validity by establishing a standardized research process: firstly, the interview outline is strictly standardized, and all questions are developed based on previous research and combined with the purpose of this study to ensure clear and unambiguous question expression; Implement structured control during the interview process to avoid deviating from the research topic; Standardize transcription and coding of interview materials in the later stage.

The above operation process provides reliable reliability and validity support for the interview results.

### 3.3 Result

Following a Strauss and Corbin style grounded theory procedure (open coding → axial coding → selective coding), we conducted an inductive, constant-comparative analysis to build an emergent conceptual model of how self-efficacy relates to high school students’ mental health. The analytic procedure was implemented in six traceable steps:

1. Familiarization and memoing. Two researchers repeatedly read the transcripts to gain an overall sense of participants’ accounts and wrote analytic memos to capture early impressions, recurring patterns, and potential theoretical directions. Memos were continuously updated throughout the analysis as an audit trail of analytic decisions.
2. Open coding. We conducted line-by-line open coding to label meaningful text segments using low-inference codes that stayed close to participants’ language (including *in vivo* codes when appropriate). Each segment could be assigned multiple codes if it conveyed more than one meaning. NVivo was used to store transcripts, attach codes, and retrieve coded excerpts efficiently.
3. Constant comparison and code refinement. Open codes were iteratively compared across participants and across interview sections to refine code boundaries, merge redundant codes, split overly broad codes, and clarify code definitions. Codebook revisions were documented in memos, and examples/decision rules were added to reduce ambiguity.
4. Axial coding. We then grouped conceptually related open codes into higher-order categories by examining conditions, context, action/interaction, and consequences. This step aimed to move beyond listing themes toward explaining relationships among categories (e.g., how specific task contexts shape fluctuations in self-efficacy and subsequent emotional/cognitive/behavioral responses).
5. Selective coding. Finally, we identified a core storyline that integrated the major categories and organized them into a coherent framework. In this study, the coding structure converged on four overarching aspects—mental health status, immediate experience of self-efficacy, long-term construction of self-efficacy, and the impact of self-efficacy on mental health—which together formed the proposed relationship model.
6. Saturation check. We monitored the emergence of new codes and categories during analysis and conducted a saturation check by re-examining later transcripts against the evolving codebook. Saturation was considered reached when no substantively new codes or category relationships emerged and when the existing framework sufficiently accounted for the data patterns.

Quantitatively, we extracted 269 semantic units (reference points) from the corpus, which were condensed into 25 first-level codes during open coding, further synthesized into 8 s-level categories during axial coding, and ultimately integrated into the four core aspects during selective coding. For transparency, Table 1 provides representative quotations for major codes; additional quotations and the full coding tree can be provided in an appendix to strengthen traceability. Due to space limitations, Table 1 will only list one reference point for each initial concept.

**Table 1:** Coding results of the impact of self-efficacy on the mental health of high school students.

Secondary encoding	First level coding	Reference point	Sample interview content
Self evaluation status of mental health	Good	5	I think I can arrange my study tasks well. At first, I found math functions difficult, so I found more practice questions to do and even watched online courses. Gradually, I mastered them. This feeling of overcoming difficulties makes me feel quite confident. Over time, the confidence gained from my studies will also affect other aspects of me.
	General	8	I think I am at a moderate level. Sometimes I think a lot about studying and exams, but these issues are not serious enough to affect my life. It’s just that they occasionally make me feel bad. I am also trying to adjust, but I have not found a particularly effective solution yet.
	Poor	10	I often cannot sleep at night and my mind is in a mess. I feel very lonely and depressed. My parents do not understand me. They always criticize me, saying that I cannot do this or that, and I cannot communicate with them.
	Self-awareness	18	I quite understand myself. I tend to have an outgoing personality, but sometimes I can be a bit impatient. For example, during group discussions, when there are too many ideas, I tend to rush to speak up. But when I realized my own problem, I was gradually making changes.
	Emotional stability	17	I am usually quite emotionally stable. But I cannot handle some bad situations. For example, if I did not do well in the last exam, I would definitely feel lost for a while. Although I will not be stuck in that emotion all the time, it will still have an impact on me. I still hope to be more stable and not constantly engage in internal conflicts
Ideal state of mental health	Self efficacy	13	Take learning as an example, I believe I can learn well. Before, I did not do well in physics and electricity, so I spent more time practicing and asking my teacher, gradually mastering the methods. Later, my exam scores improved, and ever since then, I have felt that ‘as long as I study with my heart, there is nothing I cannot learn’. This has also become my belief when encountering difficulties.
	Interpersonal relationship	12	I envy people who get along very well with their classmates. During break, we chat and joke together, but I find it difficult to integrate myself.
	Adaptability	6	When I first started high school, the difficulty of the courses increased and I felt a bit uncomfortable, resulting in a decline in my grades. But there are also middle school classmates who went to the same high school as me, some of whom adapted well. Even though they did not study as well as me before, they are now a bit ahead of me. Is it because my own adaptability is too poor.
	Examination	21	Once I took a math exam, many questions were uncertain, and there wasn’t enough time to continue, so I did not have the confidence to do them.
	Competition	7	When playing school basketball, having a good hand feel will boost one’s confidence and make their shots more accurate than usual.
Immediat e Events	Motion	5	Before learning swimming, I thought it was easy, but as soon as I got into the water, I choked and became afraid of the water. I learned very slowly.
	Speech	2	The first time I gave a speech in front of the whole class, I was so nervous that I forgot my words. It was particularly awkward at the time, but later on, I became very anxious before each speech, worried about messing it up again.
	Study	17	I have been persistently memorizing vocabulary since March and have found that reading is not as reliant on memorization as before. Not only has my accuracy improved, but I also feel that my ability to learn English is gradually improving and I am becoming more confident in my studies.
Long term events	Hobby	6	I like drawing and I always do it at home on weekends. My art teacher said I did a good job drawing before, and last month I gathered the courage to participate in a competition and won an award. I am very happy and hope to continue painting.
	Interpersonal communication	4	I have always had a good relationship with my desk mate. We share our learning and life experiences with each other, and this stable friendship gives me a sense of security.
	Success or failure experience	16	I am not good at socializing with people. During an event, because of my personality, I did not do well and felt terrible and afraid of embarrassment. Later, I did not want to go to any club activities anymore.
Source	Persuasion from others	11	The teacher said my essay was creative and encouraged me to try submitting it. Although I was still a little scared, the teacher’s encouragement was also a recognition of myself.
	Social support	19	Once I did poorly on an exam and felt very down. When I got home feeling unhappy, my mother comforted and comforted me.
	Cognitive performance	3	When participating in the sports recruitment, I was assigned to a group that seemed to be very good at sports, but as soon as I started running, I realized why I wasn’t as fast as me. I felt like I was overthinking and should focus on myself instead of observing other classmates.
Immediat e effect	Emotional display	17	The ideas proposed in today’s group discussion were adopted by everyone, and I am very happy. I feel valuable and have a pleasant mood all day.
	Task performance	12	When taking the comprehensive science exam this time, I could not do the first questions and did not want to write the rest, resulting in a very “explosive” score in the end.
	Cognitive performance	14	I think I have quite high confidence and will not deny myself just because I did something wrong once.
Long-term effect	Emotional display	10	I am quite hardworking myself, but my grades have always been poor and I lack confidence in my studies. However, I still have some expectations for my progress, which leads to anxiety when I am in school.
	Task performance	16	In the geography class, each group was required to report on the geographical knowledge of a country in terms of humanities and nature. Although I was selected to report, the teacher praised me after the end. I am actually a bit afraid of showing off in front of everyone, but afterwards I dare to challenge myself in some things that I am not very good at.

## 4 Discussion

**Figure 1:**
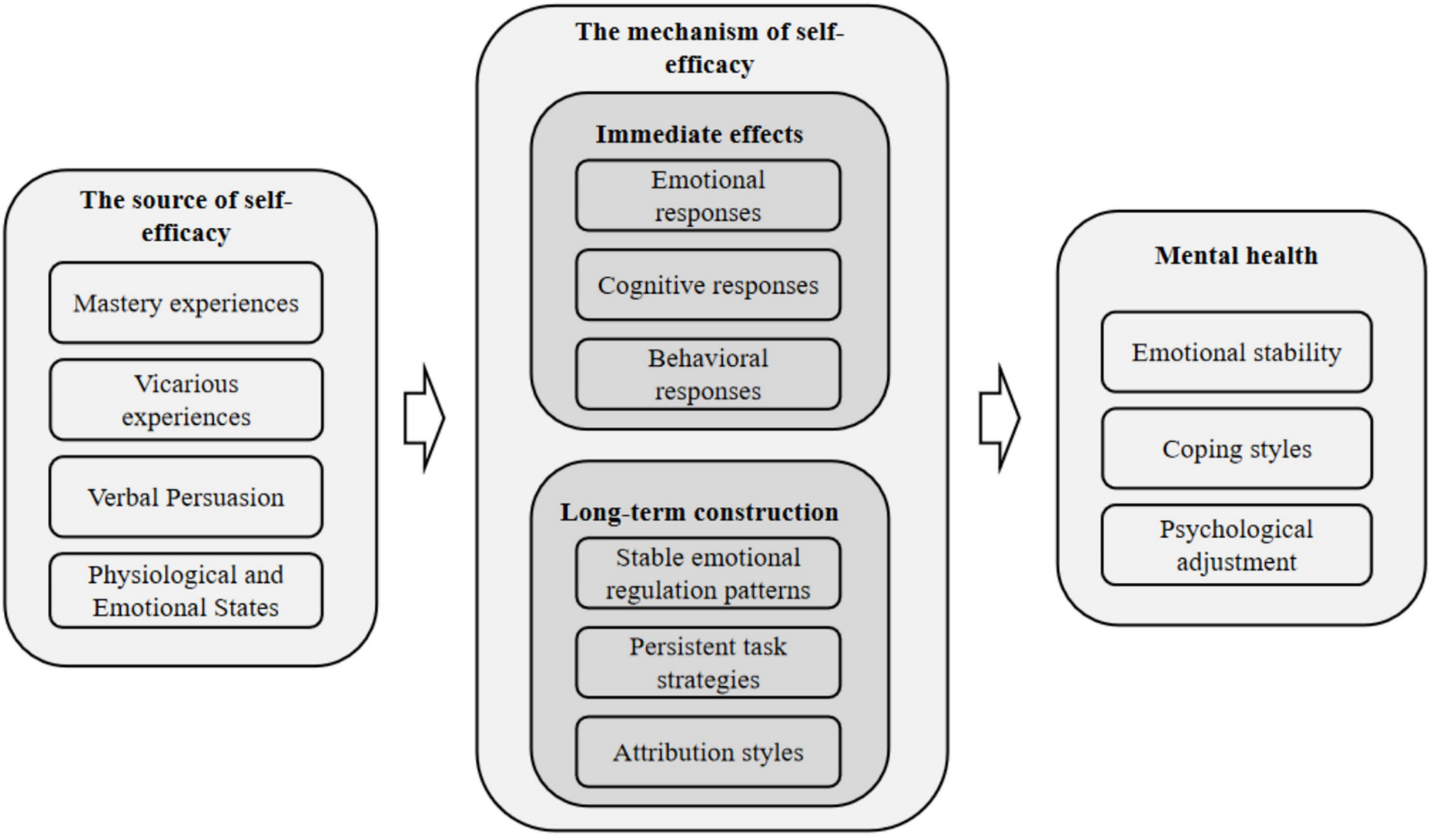
Relationship model of self-efficacy and mental health.

Building on the grounded categories reported above, Figure 1 summarizes the empirically derived mechanism linking self-efficacy and mental health.

Firstly, based on the interview results, it can be concluded that there are significant issues with the mental health status of high school students that cannot be ignored. The self-evaluation status of high school students’ mental health shows differentiated characteristics, with a certain proportion of students perceiving their psychological state as unsatisfactory, which is consistent with previous research results (Wang et al., 2021). The expected mental health status of high school students includes five parts: self-awareness, emotional stability, self-efficacy, interpersonal relationships, and adaptability. The reference point for self-awareness and emotional stability is relatively high, reflecting the internal needs of most students for clear self-awareness and stable emotions; Self efficacy is secondary to interpersonal relationships, indicating that students value and hope to improve their sense of self-efficacy and facilitate smooth interpersonal communication. Specifically, the formation of self-efficacy is based on direct experience, alternative experience, social persuasion, and physiological and emotional states (Bandura, 1977). These sources provide a clear pathway for self-efficacy to intervene in the mental health of high school students.

Secondly, starting from the immediate effect, self-efficacy often plays an important role in high school students’ exams, competitions, sports, speeches, and other activities, affecting their cognition, emotions, and task performance. Among them, emotional fluctuations are the most sensitive: when self-efficacy is low, respondents are prone to negative emotions such as sadness, frustration, and anxiety after encountering setbacks (S10; core dimension: immediate effects; category: affective response; code: anxiety triggered by low efficacy in exam preparation). High self-efficacy individuals are more likely to experience positive emotions such as happiness, pride, and competence during tasks. It should be noted that the emotions triggered by a single task are short-lived, but long-term low self-efficacy may cause high school students to experience cumulative effects of multiple immediate negative experiences, repeatedly internalizing task setbacks as self denial of insufficient ability, ultimately evolving into sustained negative emotions, posing potential risks to their mental health. The interview results showed that more than half of the respondents believed that changes in self-efficacy would directly affect their performance in subsequent tasks. When students experienced enhanced efficacy through supportive feedback, they reported a rapid shift toward approach-oriented strategies and greater willingness to engage with challenges. One participant described how teacher affirmation in class immediately increased their readiness to attempt difficult problems: after the teacher praised my correct answers in class, I wanted to try difficult questions (S7; core dimension: immediate effects; category: behavioral activation; code: social persuasion increasing challenge-seeking). Together, these excerpts support the interpretation that short-term efficacy fluctuations can produce observable changes in affect and task engagement within the same situational episode. Importantly, these immediate affective reactions may be short-lived at the single-task level; however, the interviews also indicate a risk mechanism whereby repeated low-efficacy episodes can accumulate, leading students to internalize task setbacks as evidence of insufficient ability. This repeated internalization may, over time, contribute to more persistent negative affect and heightened mental health vulnerability.

Again, the long-term effect of self-efficacy is not a simple accumulation of immediate effects, but rather the formation of a more stable psychological pattern through the interaction of cognition, emotion, and behavior. Emotionally, it manifests as the normalization of positive emotions and the rapid relief of negative emotions. High self-efficacy individuals are more likely to activate positive emotional response patterns when facing pressure. S5 respondents expressed that they are very confident in their studies, so they feel excited rather than anxious before exams (S5; core dimension: long-term effects; category: emotional regulation under stress; code: positive affect activation through high efficacy). Meanwhile, high self-efficacy enhances an individual’s ability to buffer negative emotions. When faced with setbacks, high self-efficacy individuals often focus on the problem itself rather than falling into negative emotions. In terms of task performance, self-efficacy forms a relatively stable task execution strategy for individuals through the influence of cognition and emotions. In long-term tasks, high self-efficacy individuals exhibit stronger persistence and can regulate their emotions to continue completing tasks even in the face of setbacks. A senior student facing heavy academic pressure emphasized a stable expectancy of reward from effort: I always believe my efforts will be rewarded. As long as I study seriously, my grades will definitely improve (S2; core dimension: long-term effects; category: persistence and effort; code: effort–outcome expectancy and perseverance). This suggests that long-term self-efficacy operates by stabilizing task strategies and maintaining goal-directed behavior through adverse conditions. In terms of cognitive performance, self-efficacy can trigger cognitive transformation through the continuous accumulation of experience, which is fundamentally different from cognitive fluctuations in immediate effects. High self-efficacy individuals often transfer their successful experiences in one field to other fields, gradually forming confidence across different scenarios. Low self-efficacy individuals are prone to attribute task failure to insufficient ability, which can weaken their willingness to work hard in subsequent tasks (Wu et al., 2020).

Finally, for high school students, their sense of efficacy mainly comes from areas such as academic achievement, social performance, interests, and strengths. The psychological development characteristics of this stage make the impact of self-efficacy fluctuations on their mental health more significant (Yu, 2022). Self efficacy has an impact on an individual’s mental health through the cognitive emotional behavioral linkage mechanism. From a positive perspective, individuals with high self-efficacy tend to view challenges as opportunities for skill improvement. This cognitive pattern reduces excessive anxiety about failure and lowers the likelihood of negative thoughts such as depression and self denial (Schönfeld et al., 2019).

## 5 Education suggestion

Based on the qualitative analysis and quantitative coding of the above interview results, it is urgent to construct a developmental intervention system with self-efficacy cultivation as the core in the practice of mental health education (Crosland et al., 2024). Specifically, relying on social cognitive theory, a multidimensional approach that integrates education and home school collaboration mechanisms can help high school students form a stable self-efficacy cognitive schema during the critical period of adolescent development. The construction of this system not only helps to strengthen the psychological health level of high school students in multiple aspects such as academic achievement, emotional regulation, and social adaptation, but also lays a good psychological resource foundation for their lifelong development through the cross situational transfer characteristics of self-efficacy. From a developmental perspective, it upgrades psychological health education from problem intervention to literacy cultivation (Renninger, 2010).

### 5.1 Penetration education construction guided by self-efficacy

Firstly, schools need to break the traditional psychological health education curriculum model of “knowledge lectures,” construct a three-dimensional cultivation system of “experience cognition behavior,” and attach importance to non curriculum psychological health education (Ojio et al., 2021). As an important means of mental health education, mental health education courses are an effective way for high school students to understand mental health knowledge and improve their psychological quality. However, due to the limited class hours and fixed teaching content, the role of mental health education courses in promoting the mental health level of high school students is relatively limited. At the same time, the methods and approaches adopted by non curriculum carriers will directly affect the effectiveness of high school students’ mental health education. Appropriate methods can not only stimulate students’ learning initiative, but also achieve effects that curriculum education cannot achieve. Therefore, through multi-dimensional collaboration of school management, activities, and environment, a multi scenario and immersive psychological support ecosystem can be constructed to cultivate students’ self-efficacy and strengthen the penetration of non curriculum mental health education. Specifically, in terms of daily management, schools should increase their emphasis on mental health education, incorporate mental health indicators into school development plans, and collaborate with other departments to create a cultural atmosphere of concern for mental health throughout the school (Zhang, 2016). Ultimately, this will lead to a transformation of mental health education from passive intervention to active development. In terms of activities, increase mental health themed activities and experiential psychological intervention activities, enrich campus activity forms, and promote students’ physical and mental development together. In terms of environmental creation, the physical environment can be designed and transformed based on colors, sounds, and even students’ ideas to create a campus environment; the psychological environment can be constructed through language campus broadcasting, growth rituals, cultural symbols, and other aspects (La Salle et al., 2021).

Secondly, schools should integrate efficiency education into the multidisciplinary teaching process. The core of efficacy education is to cultivate students’ self-efficacy, that is, to make them believe that they have the ability to complete learning tasks, which is manifested in the formation of efficient learning strategies at the cognitive level; Enhance learning confidence and reduce frustration on an emotional level; At the behavioral level, it manifests as the ability to actively plan learning and independently solve problems (Lin, 2021). Therefore, in order to break through the traditional teacher training of skill imparting, it is necessary to build a cognitive skill emotional empowerment system, achieve the coordinated development of teacher and subject effectiveness education, and provide new practical paths for the cultivation of students’ mental health. Teacher effectiveness training not only enhances one’s own belief in teaching effectiveness, but also integrates effectiveness education into subject teaching, achieving a transformation from a knowledge transmitter to a psychological enabler. By setting goals, guiding the process, and providing feedback through evaluation, we aim to enhance students’ sense of learning efficacy and comprehensive literacy, in order to achieve the goals of efficacy education mentioned above. Specific methods include target visualization, precise feedback, and exemplary demonstration. Goal visualization is to allow students to see their progress trajectory. During the teaching process, set performance goals for each unit, such as mastering 100 English words this week and providing a goal achievement checklist. Students can record their completion status at any time and enhance their confidence through visual progress. Precision feedback refers to the use of specific praise by educators to enhance effectiveness, avoiding vague evaluations, such as changing from “you are great” to “you solved the problem using a different method today, which is very efficient,” allowing students to clarify their strengths and strategies, and strengthen their sense of self-efficacy. Role model demonstration is the transmission of efficacy beliefs through peer cases, making the successful experiences of peers replicable alternative experiences. Penetrating efficiency education into the process of individual knowledge formation not only promotes individual mental health, but also cultivates positive psychological qualities.

Finally, it is necessary to strengthen the construction of self-efficacy oriented activities in high school mental health education. It is necessary to go beyond scattered activity designs, form a systematic intervention system based on the laws of developmental psychology and educational technology innovation, and create school-based and classroom activities that emphasize students’ observation, feelings, and experiences. By constructing a macro cognitive framework through school-based activities and shaping micro behavioral patterns through classroom activities, we continuously strengthen our sense of self-efficacy, internalize external efficacy experiences into stable psychological qualities, and ultimately achieve a deep transformation from activity participation to personality growth, thereby fundamentally promoting the formation and development of a sound personality. In short, by integrating self-efficacy into mental health education through the above methods, mental health education is no longer an independent task of the subject, but a natural tool for students to understand knowledge, develop thinking, and adjust their psychology (Zhang et al., 2024).

### 5.2 Home school collaboration promotes high school students’ mental health

General Secretary Xi Jinping clearly pointed out at the 2024 National Education Conference that in order to run education well, families, schools, governments, and society are indispensable. Family is the first school in life, and parents are the first teachers of their children. School education and family education essentially form an organic unity that promotes the development of young people. As the core elements of students’ growth process, the two play an indispensable role in the physical and mental development and comprehensive quality improvement of students through the coordinated resonance of educational goals and the complementary integration of educational resources. Based on the integration framework of social cognitive theory and ecosystem theory, home school collaboration is an important way to promote the mental health development of high school students (Miller et al., 2021). It requires theoretical anchoring in the cognitive dimension, technical construction in the methodological dimension, and data empowerment in the technical dimension to promote the mental health development of high school students, which includes two specific paths.

Firstly, schools need to reconstruct parental cognition and highlight the importance of cultivating self-efficacy (Xue et al., 2023). When parents rely on empirical or fragmented information to form cognitive frameworks, it can lead to systematic biases in their assessment of their children’s psychological states. The reconstruction of parental self-efficacy cultivation cognition is essentially a paradigm shift in educational philosophy from outcome oriented to growth oriented. Schools need to systematically help parents establish self-efficacy as the core cognitive ability of psychological development through lectures, class meetings, and other means, ultimately forming a cognitive resonance and action resonance between family and school in efficacy cultivation, and building a sustainable psychological resource system for students. This process requires schools to play a professional leading role in transforming cognitive reconstruction into actionable, assessable, and iterative system engineering, rather than scattered promotional and educational activities.

Secondly, based on the interview results of this study and social cognitive theory, a family school collaborative support system is constructed (Lu and Yu, 2024). On the one hand, based on social cognitive theory, a family scenario efficacy intervention tool system is constructed by school psychology professionals. The system is designed with ecological intervention as the logic, integrating operational modules such as achievement experience, alternative experience transfer, and growth oriented language communication. Through structured tools, parents can cultivate high school students’ self-efficacy in the family environment (Xu et al., 2023). On the other hand, the use of efficacy assessment tools by parents is also of great significance for class teachers to understand students’ psychological dynamics. It not only helps schools integrate students’ psychological conditions in their daily lives, but also establishes a behavioral indicator system for the development of students’ efficacy, realizes the normalization monitoring and early warning of individual psychological states, and forms a closed-loop intervention system for efficacy cultivation between home and school. This not only ensures that the parent group can learn scientific and effective intervention strategies, but also provides an assessment of students’ psychological status for class teachers, achieving synergy between family support and school intervention. Furthermore, schools can develop a digital home school psychological platform to break through the temporal and spatial limitations of traditional home school collaboration. Through the digital transformation of efficiency training tools, the psychological health intervention model can be upgraded, ultimately forming a modern psychological education ecosystem that provides full cycle, cross scenario digital support for the psychological health development of high school students (Balcombe and De Leo, 2022).

The above two methods have solved the problem of only sharing experiences between teachers and parents in the traditional home school collaboration process. Establish a specialized collaborative system based on social cognitive theory and supported by digital technology. Especially by expanding the cultivation of self-efficacy from a single psychological intervention to a systematic engineering of cognitive reconstruction, behavioral training, and digital ecological construction, it enriches the ways of adolescent mental health education.

## Data Availability

The original contributions presented in the study are included in the article/supplementary material, further inquiries can be directed to the corresponding author.
